# A Purkinje image-based system for an assessment of the density and transmittance spectra of the human crystalline lens in vivo

**DOI:** 10.1038/s41598-020-73541-y

**Published:** 2020-10-05

**Authors:** Taisuke Eto, Petteri Teikari, Raymond P. Najjar, Yuki Nishimura, Yuki Motomura, Manami Kuze, Shigekazu Higuchi

**Affiliations:** 1 Graduate School of Integrated Frontier Sciences, Kyushu University, Fukuoka, Japan; 2 Research Fellow of the Japan Society for the Promotion of Science, Fukuoka, Japan; 3 UCL Queen Square Institute of Neurology, University College London, London, UK; 4 Department of Visual Neuroscience, Singapore Eye Research Institute, Singapore, Singapore; 5 The Ophthalmology & Visual Sciences ACP (EYE-ACP), SingHealth and Duke-NUS Medical School, Singapore, Singapore; 6 National Institute of Occupational Safety and Health, Kawasaki, Japan; 7 Department of Human Science, Faculty of Design, Kyushu University, 4-9-1 Shiobaru, Minami-ku, Fukuoka, 815-8540 Japan; 8 Ophthalmology Clinic, Matsusaka Central General Hospital, Matsusaka, Japan; 9 Department of Ophthalmology, Mie University Graduate School of Medicine, Tsu, Japan

**Keywords:** Lens diseases, Ageing

## Abstract

A method for rapid and objective assessment of ocular lens density and transmittance is needed for research and clinical practice. The aim of this study was to determine whether the Purkinje image-based technique can be used for objective and accurate quantification of spectral density and transmittance of ocular media (the mainly crystalline lens) in visible light. Twenty-six individuals (10 young, 9 middle-aged and 7 older individuals) participated in this study. Spectral lens density was evaluated by detecting the intensity of the IVth Purkinje image for different wavelengths. Subsequently, optical density index (ODI), the area under the curve in the lens density spectrum, was calculated and ODIs were compared with clinical lens opacification scales assessed subjectively using a slit lamp. Spectral lens transmittance was estimated from the lens density spectrum. Lens densities were higher in the short wavelength region of the visible spectrum across all age groups. ODI was highly correlated with the clinical opacification scale, while lens transmittance decreased with aging. Our results showed that spectral transmittance of the human crystalline lens can be easily estimated from optical density spectra evaluated objectively and rapidly using the Purkinje image-based technique. Our results provide clinicians and scientists with an accurate, rapid and objective technique for quantification of lens transmittance.

## Introduction

Aging is associated with an increase in spectral optical density and a concomitant decrease in transmittance of the human crystalline lens, especially in the short wavelength blue visible region of the light spectrum^1–3^. This age-related change in the crystalline lens, affects various visual^4–6^ and non-visual functions^7–12^ as it alters the intensity and spectral composition of the light information reaching retinal photoreceptors. In addition, the increase of optical density can lead to the formation of cataracts, the main cause of reversible blindness worldwide^13^. Therefore, the objective assessment of the crystalline lens’s spectral optical density and transmittance is valuable for the research and clinical settings.

The measurements of the spectral optical density and transmittance of the human crystalline lens were initially and predominantly conducted on extracted donor lenses^3,14–19^. Methods for measuring the spectral lens density and transmittance in vivo have also been studied^20–24^. Recently, Teikari et al*.*^25^ demonstrated that spectral transmittance of the lens can be estimated from optical density measured via a scotopic heterochromatic flicker photometry (sHFP) method, using the ocular media model proposed by van de Kraats and van Norren^26^. Subsequently, Najjar et al. verified that lens density measured using sHFP correlates with clinical evaluation and yields a better predictor of increase in lens density with aging than other physical and psychophysical methods^27^. However, psychophysical methods such as sHFP are difficult to implement for individuals with visual impairments or children as these procedures are lengthy and rely on the subject’s feedback, visual perception and fixation aptitudes.

In contrast to these psychophysical methods, alternate direct measurements have been studied^28–32^. Said and Weale measured lens density spectra by a physical method based on Purkinje images^33^. Purkinje images, formed by reflections of light sources at different ocular interfaces (Ist Purkinje image: air-cornea, IInd: cornea-aqueous humor, IIIrd: aqueous humor-lens and IVth: lens-vitreous humor), have been used for a long time^34^. Said and Weale determined the ratio of intensities for the IIIrd and IVth Purkinje image reflections from the front and rear surfaces of the lens, respectively, for different wavelengths. Their method was applied to Sakanishi’s approach in which relative lens transmittance spectra can be calculated from the intensity ratio between IIIrd and IVth Purkinje images^35^. Unlike the psychophysical methods, it is possible that these Purkinje image-based techniques can be applied to individuals with visual impairments because these techniques do not rely on the subject’s decision. However, these techniques were time-consuming and inconvenient because the pupil needs to be dilated with pharmacological agents.

As another approach for assessment of the optical property of the lens using Purkinje images, Santos et al. proposed a technique for objective and independent assessment of light scattering caused by the cornea and the lens^36^. This technique is based on analysis of the contrast of the IIIrd and IVth Purkinje images generated by incident near-infrared (> 760 nm) light. Santos’s technique may enable assessment of spectral light scattering of the lens in visible light if the wavelength region of the light source is extended into the visible light region; however, it does not mean that spectral lens density can be measured. In addition, these methods including Said and Weale’s technique need information of the IIIrd Purkinje image, but detection of that in the visible light region is difficult because the IIIrd image is generally diffuse^34,37^ and blurry and there is possibility that this phenomenon is especially pronounced at a shorter wavelength by large light scattering^38^.

A lens absorption monitor (LAM) technique developed by Johnson et al. based on Said and Weale’s method can rapidly and objectively measure spectral optical density of the anterior segment (mainly crystalline lens) in the living human eye without the III^rd^ Purkinje image^39^. The LAM technique is based on a comparison of the intensity of the IVth Purkinje image with an external reference (the reflection of the light source off of a spectrally flat black glass bead) for different wavelengths of visible light. The IVth Purkinje image is much easier to locate than the IIIrd image, and it can usually be detected without having to dilate a pupil. According to Johnson’s report, with the LAM technique, a single set of measurements can be obtained in 2 s per eye without pupil dilatation. Although the spectral optical density of the anterior segment measured by the LAM technique consists of not only lens density but also cornea and aqueous humor density, previous studies indicated that the cornea and aqueous humor show negligible and relatively nonselective optical density of different wavelengths in the visible region^14,26,40,41^. Therefore, it can be considered that lens density spectra can be measured by the LAM technique. Despite the fact that the Purkinje image-based technique like the LAM technique, offering rapid, objective and accurate measurement of the lens density, has the possibility of being useful in research and clinical fields, there has been no report on this technique since the improvement in quality reported by Savage et al.^42^. Moreover, full light spectral transmittance can be estimated from the spectral optical density obtained by the Purkinje image-based procedure by fitting with the ocular media model^26^ based on Teikari’s approach^25^.

The aim of this study was to determine the feasibility of a system that can quickly, objectively and accurately measure the human lens density spectrum and estimate the lens transmittance spectrum in vivo. As the first step, we revaluated the accuracy of lens density measured by the Purkinje image-based technique. We investigated whether measurements of lens density using the Purkinje image-based technique (1) show wavelength dependence (i.e., increase in lens density in a short wavelength) and age-related increase of lens density as found in previous studies^1,2,39,42^; (2) correlate with clinical assessments of lens-status using a slit lamp and (3) are affected by pupil size (non-mydriasis or mydriasis). After confirming the accuracy of the Purkinje image-based technique, we investigated whether a lens transmittance spectrum can be estimated from the lens density spectrum using the ocular media model proposed by van de Kraats and van Norren^26^.

## Results

### Optical density spectra of lenses measured by the Purkinje image-based technique and relationships with age and results of subjective assessment

Twenty-six participants were included in this study (age range 22–67 years; mean age ± SD, 40.7 ± 12.8 years). Participants were divided into a young group (n = 10; 20–34 years old; 27.2 ± 3.7 years old), a middle-aged group (n = 9; 35–49 years old; 42.7 ± 4.1 years old) and an older group (n = 7; 50–70 years old; 58.9 ± 6.7 years old). A single set of measurements by our Purkinje image-based technique was performed in approximately 4 s per eye.

**Figure 1:**
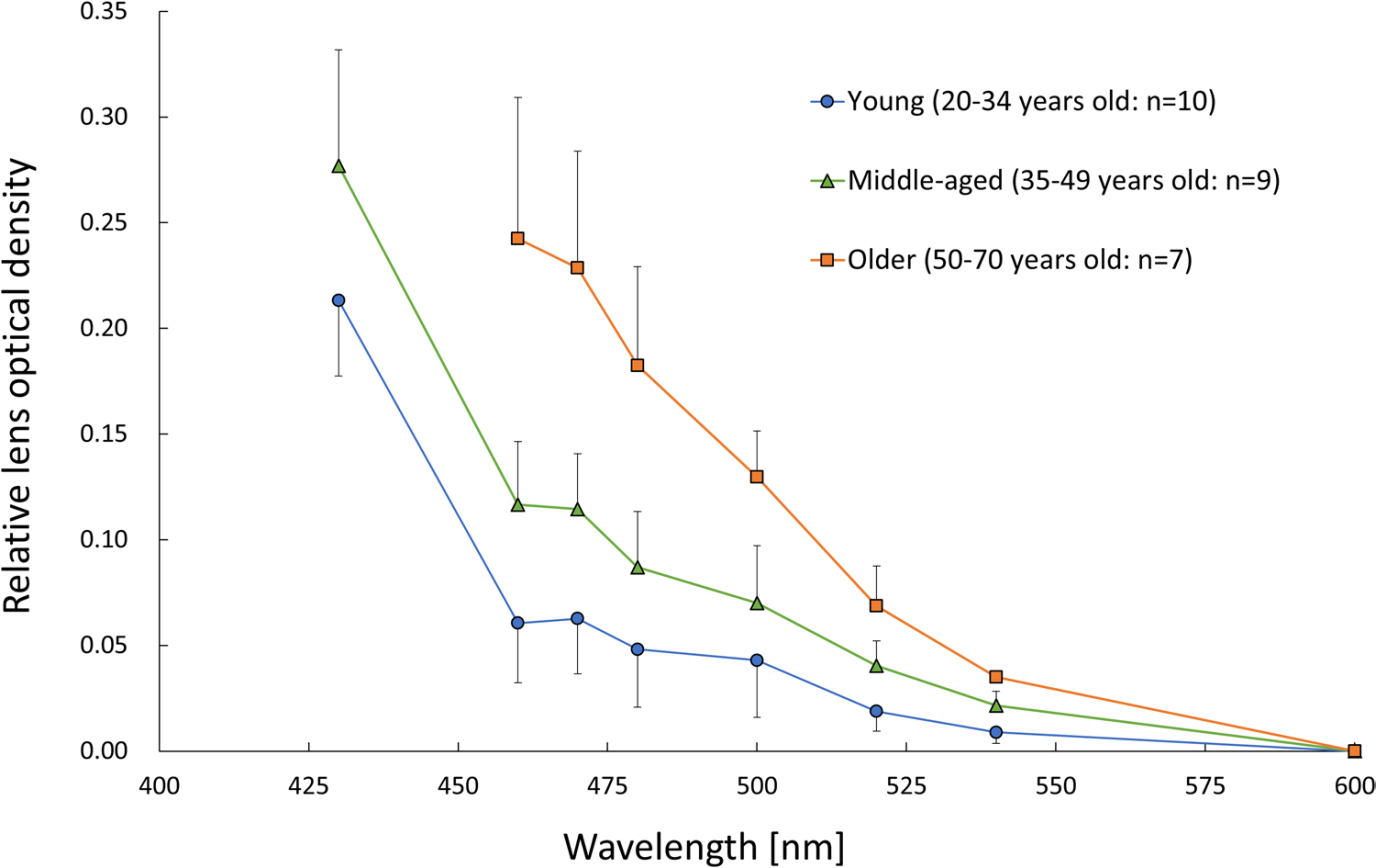
Average spectral optical density of the crystalline lens obtained by using the Purkinje image-based technique for each age group (young group: mean − SD, middle-aged and older group: mean + SD). Results for the young group are shown as blue circles and a blue line, those for the middle-aged group are shown as green triangles and a green line, and those for the older group are shown as orange squares and an orange line.

**Figure 2:**
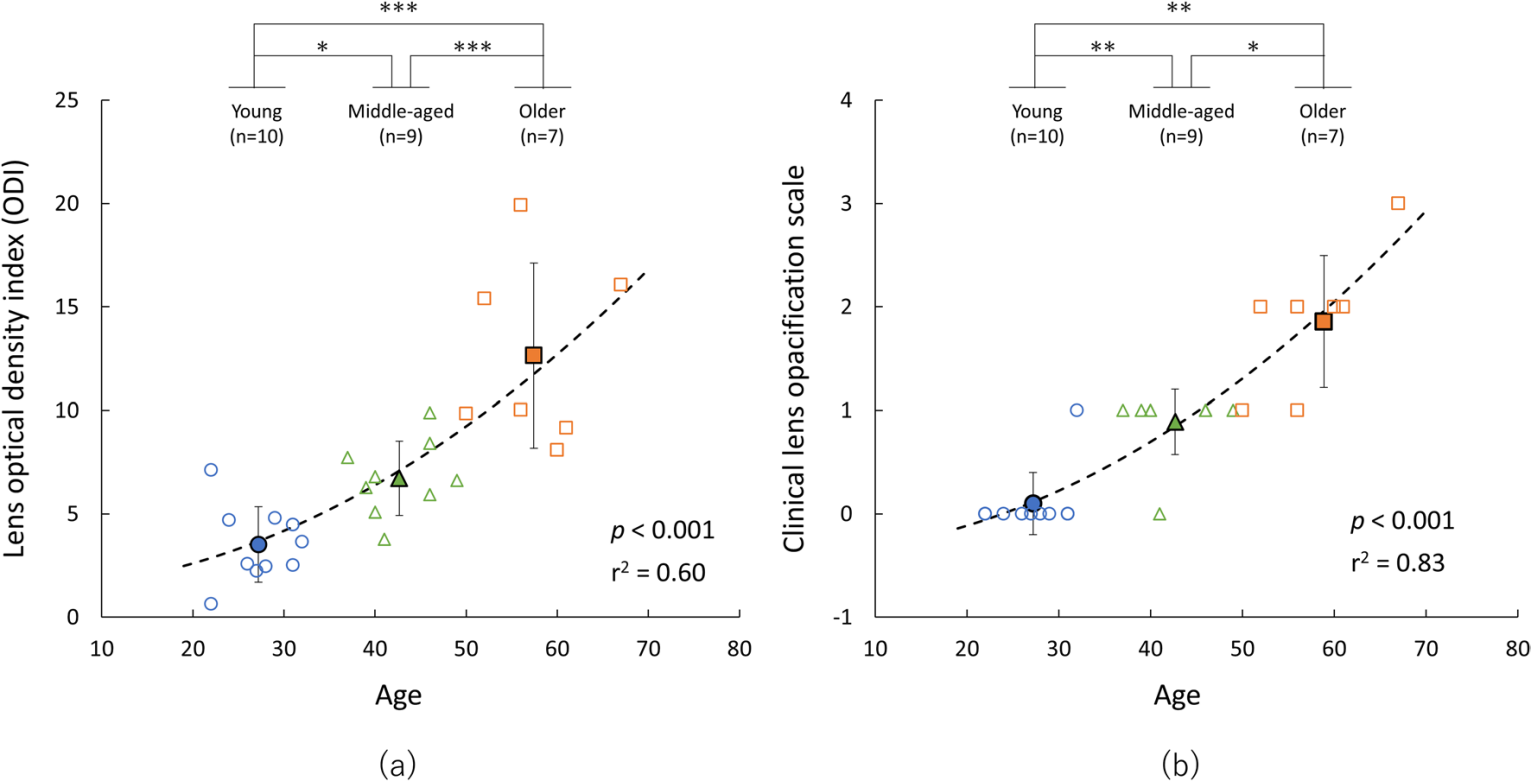
Individual lens density (ODI and opacification scale) as a function of age. Lens density was assessed by using (**a**) the Purkinje image-based technique and (**b**) a clinical ophthalmologic slit beam procedure. Individual lens opacity is shown as colored opened symbols. Results for the young group are shown by blue circles, those for the middle-aged group are shown by green triangles, and those for the older group are shown by orange squares. Lens density was fitted by using a quadratic trend that ignores the linear term, and the regression line is shown by a black dashed line. Average lens opacity (mean ± SD) of each age group is shown by colored filled symbols on top of the individual scatter plot. Results for the young group are shown by blue circles, those for the middle-aged group are shown by green triangles, and those for the older group are shown by orange squares. ***P < 0.001, **P < 0.01, *P < 0.05.

**Figure 3:**
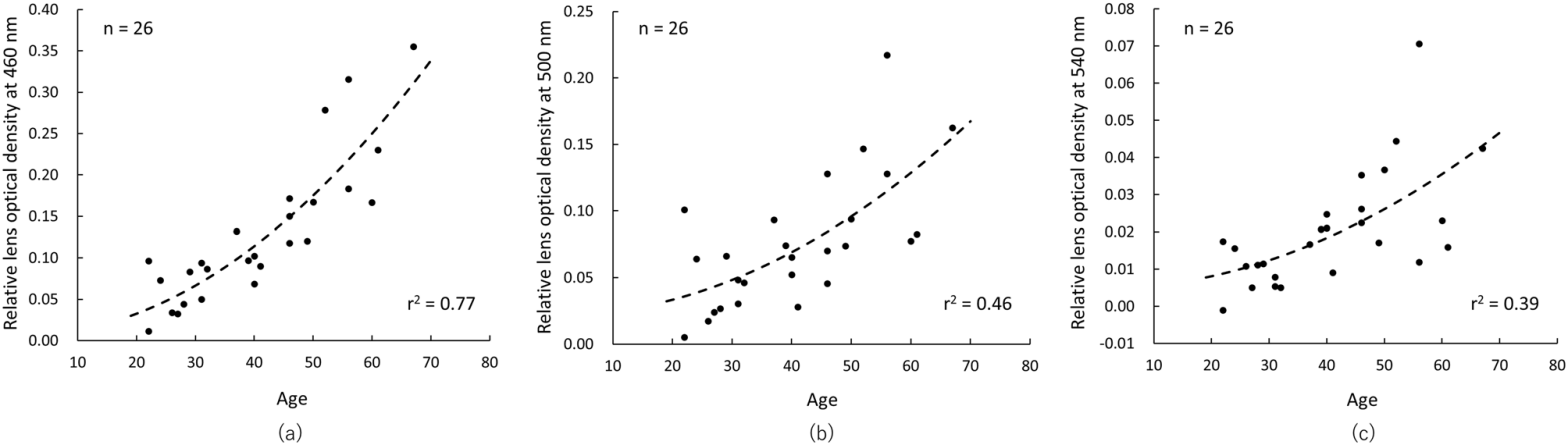
Individual lens optical densities measured by the Purkinje image-based technique as a function of age at (**a**) 460, (**b**) 500 and (**c**) 540 nm. Optical density was fitted by using a quadratic trend that ignores the linear term, and the regression line is shown by a black dashed line.

**Figure 4:**
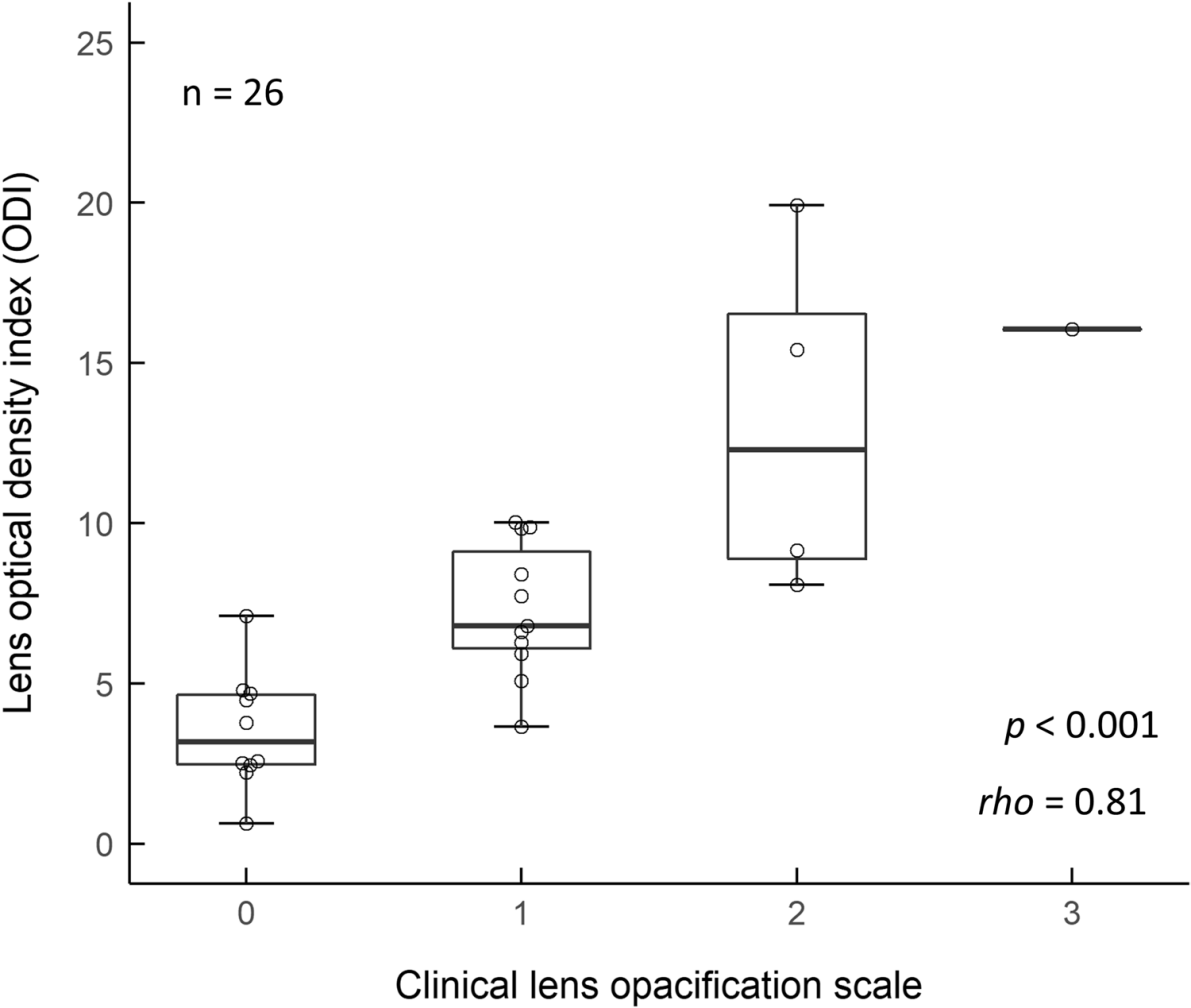
Comparison of lens densities determined by the Purkinje image-based technique (optical density index: ODI) and by the clinical subjective method (lens opacification scale).

The optical densities measured by the Purkinje image-based technique were higher as the wavelength became shorter in all age groups (main effects of wavelength: young group [*F* (2.94, 26.48) = 134.51, *P* < 0.001, *η*^2^ = 0.94], middle-aged group [*F* (2.31, 18.52) = 137.43, *P* < 0.001, *η*^2^ = 0.95], and older group [*F* (1.81, 10.86) = 61.83, *P* < 0.001, *η*^2^ = 0.91]) (Fig. 1). In the results for optical density index (ODI), which is the area under the curve of the optical density spectrum in the range from 460 to 600 nm, one-way ANOVA showed a significant main effect of age (*F* (2, 23) = 22.55, *P* < 0.001, *η*^2^ = 0.66). Post hoc analyses revealed that ODI in the older group was significantly higher than ODIs in the middle-aged group (*P* < 0.001) and the young group (*P* < 0.001) and that ODI in the middle-aged group was significantly higher than that in the young group (*P* = 0.019) (Fig. 2a). In results for the clinical lens opacification scale, the Kruskal–Wallis test showed a significant main effect of age (*χ*^2^ = 19.2, *P* < 0.001). Post hoc analyses revealed that the opacification scale in the older group was significantly higher than these in the middle-aged group (*P* = 0.014) and the young group (*P* = 0.001) and that the opacification scale in the middle-aged group was significantly higher than that in the young group (*P* = 0.003), the same as the results for ODI (Fig. 2b). The ODI and clinical lens opacification scale were highly correlated with age (quadratic trend, ODI: *r*^2^ = 0.60, *P* < 0.001, opacification scale: *r*^2^ = 0.83, *P* < 0.001) (Fig. 2a,b). Regarding the relationship between age and optical densities measured by the Purkinje image-based technique at 460, 500 and 540 nm, optical density at 460 nm was more highly correlated with age (*r*^2^ = 0.77) than was optical density at 500 (*r*^2^ = 0.46) or 540 nm (*r*^2^ = 0.39) (Fig. 3). In addition, ODI was highly correlated with clinical lens opacification scale (*P* < 0.001, *rho* = 0.81) (Fig. 4).

### Effect of pupil condition on measurements by the Purkinje image-based technique

**Figure 5:**
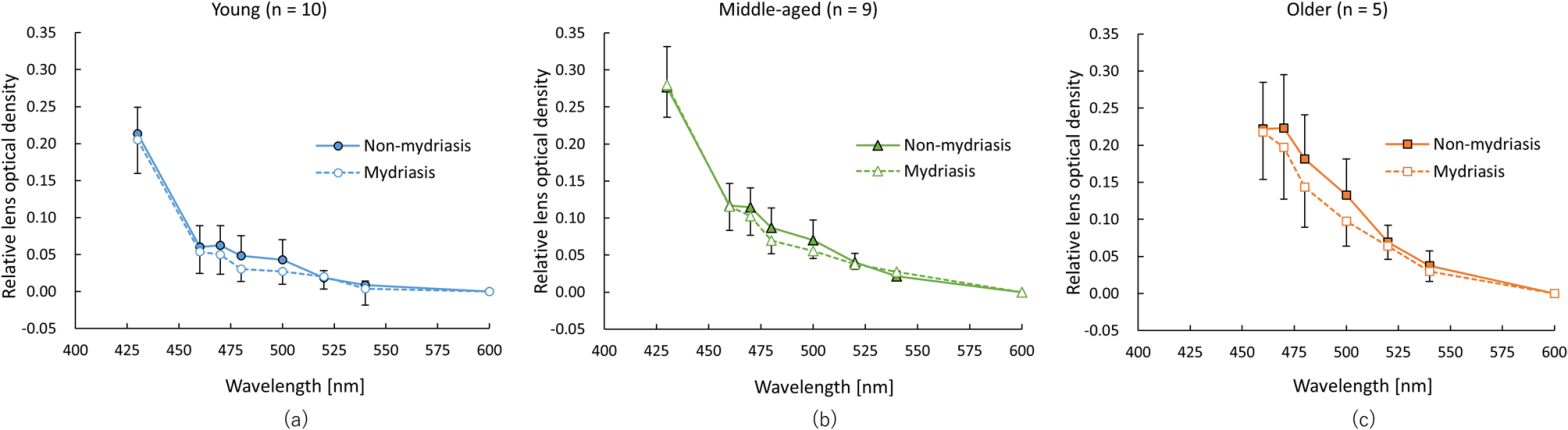
Effect of pupil condition (non-mydriasis or mydriasis) on optical density measured by the Purkinje image-based technique. In the young group (**a**), optical density in a non-mydriasis condition is shown by blue filled circles (mean + SD) and a blue solid line and optical density in a mydriasis condition is shown by blue opened circles (mean − SD) and a blue dashed line. In the middle-aged group (**b**), optical density in a non-mydriasis condition is shown by green filled triangles and a green solid line and optical density in a mydriasis condition is shown by green opened triangles and a green dashed line. In the older group (**c**), in a non-mydriasis condition is shown by orange filled squares and an orange solid line and optical density in a mydriasis condition is shown by orange opened squares and an orange dashed line.

Mixed repeated measures ANOVA for investigating the effect of pupil size on optical density measured by the Purkinje image-based technique revealed a significant main effect of wavelength in all age groups (young group: *F* (2.3, 41.46) = 271.88, *P* < 0.001, *η*^2^ = 0.68, middle-aged group: *F* (2.17, 34.73) = 325.04, *P* < 0.001, *η*^2^ = 0.71, older group: *F* (1.65, 16.52) = 84.0, *P* < 0.001, *η*^2^ = 0.62), but there was no significant main effect of pupil condition (young group: *F* (1, 18) = 0.01, *P* = 0.92, *η*^2^ < 0.001, middle-aged group: *F* (1, 16) = 1.62, *P* = 0.22, *η*^2^ = 0.02, older group: *F* (1, 10) = 0.0009, *P* = 0.98, *η*^2^ < 0.001) and there was no significant interaction between wavelength and pupil condition (young group: *F* (2.3, 41.46) = 0.62, *P* = 0.56, *η*^2^ = 0.002, middle-aged group: *F* (2.17, 34.73) = 0.78, *P* = 0.48, *η*^2^ = 0.002, older group: *F* (1.65, 16.52) = 0.80, *P* = 0.45, *η*^2^ = 0.006) (Fig. 5). The pupil sizes with and without mydriasis during measurements by the Purkinje image-based technique in each age group are shown in Table 1.

**Table 1:** Size of pupils without and with mydriasis during measurements by the Purkinje image-based technique in each age group.

Wavelength (nm)	Young group (n = 10)		Middle-aged group (n = 9)		Older group (n = 5)	
	Mean ± SD (mm)		Mean ± SD (mm)		Mean ± SD (mm)	
	Non-mydriasis	Mydriasis	Non-mydriasis	Mydriasis	Non-mydriasis	Mydriasis
430	6.14 ± 0.78	7.80 ± 0.48	5.24 ± 0.68	7.38 ± 0.37	4.70 ± 0.68	6.96 ± 0.48
460	5.27 ± 0.77		4.47 ± 0.64		4.06 ± 0.64	
470	4.88 ± 0.85		4.24 ± 0.69		3.86 ± 0.65	
480	4.71 ± 0.83		4.18 ± 0.77		3.89 ± 0.69	
500	4.56 ± 0.85		4.14 ± 0.78		3.93 ± 0.72	
520	4.40 ± 0.86		4.03 ± 0.71		3.87 ± 0.70	
540	4.28 ± 0.83		3.92 ± 0.71		3.70 ± 0.61	
600	4.24 ± 0.79		3.84 ± 0.77		3.55 ± 0.59	

### Estimation of lens transmittance spectra from measurements by the Purkinje image-based technique

**Figure 6:**
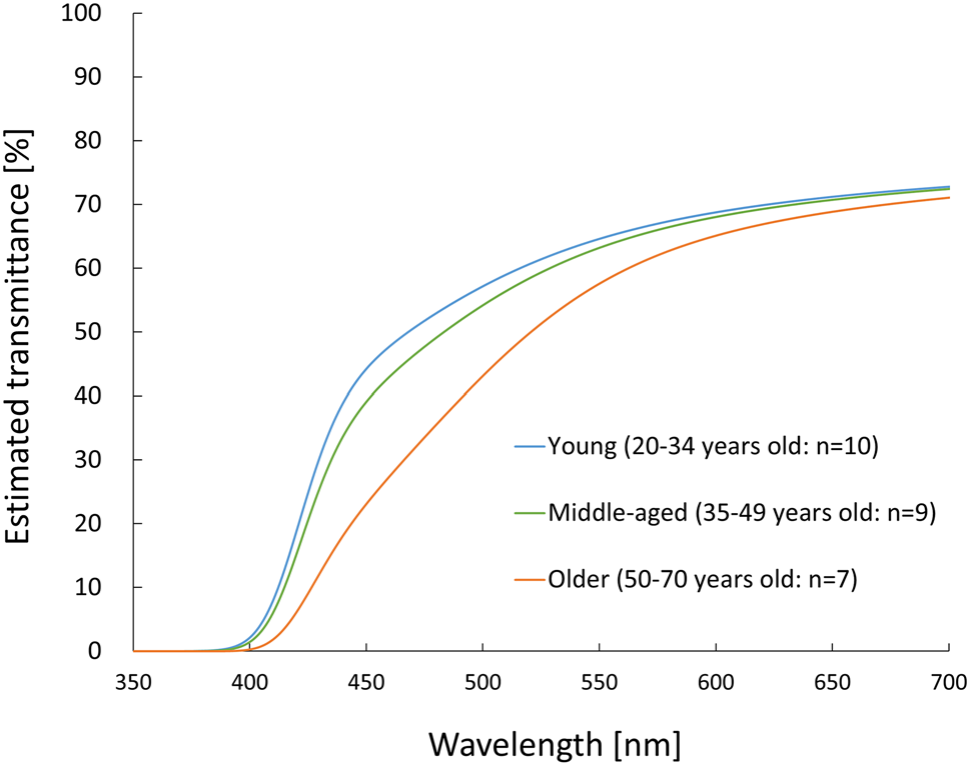
Lens transmittance spectra of the crystalline lens estimated by the Purkinje image-based technique. The estimated transmittance spectrum in the young group is shown by a blue line, that in the middle-aged group is shown by a green line, and that in the older group is shown by an orange line.

Figure 6 shows the estimated spectral transmittance from optical density measured by the Purkinje image-based technique using the ocular media model proposed by van de Kraats and van Norren^26^. Transmittance spectra in the age groups of participants were compared. One-way ANOVA showed a significant main effect of age on AUC of transmittance spectra (*F* (2, 23) = 22.97, *P* < 0.001, *η*^2^ = 0.67). Transmittance was significantly lower in the older group than in the middle-aged group (*P* < 0.001) and the young group (*P* < 0.001). However, there was no significant difference in transmittance between the middle-aged and the young group (*P* = 0.08).

## Discussion

Our results demonstrated that ODI, which is an objective index for optical density of the human crystalline lens measured in approximately 4 s by the Purkinje image-based technique, is a useful index for detecting age-related changes of the lens with a high level of accuracy. Moreover, the results for spectral lens density showed that the increase in spectral lens density with aging is particularly pronounced for short wavelength light as found in previous studies^1,2^. We also confirmed that spectral lens transmittance estimated from spectral lens density measured by the Purkinje image-based technique decreased with aging, and these results are in line with results of previous donor^3,18,19^ and in vivo^25,27,30^ studies.

Savage et al. reported that the average lens densities measured by the Purkinje image-based technique at 430 nm in 24-year-old and 50-year-old (average ages) subjects were 0.20 and 0.39, respectively^42^. In our study, lens densities measured at 430 nm in a 24-year-old subject and a 50-year-old subject were 0.1956 ≈ 0.20 and 0.3854 ≈ 0.39, respectively. Although the results reported by Savage et al. were average values, our respective findings are in agreement and suggest that the Purkinje image-based technique in highly reproducible.

In our study, we also show that ODI predicted with a relatively high level of accuracy in quadratic trend^26^ the increase in lens density with aging (*r*^2^ = 0.60). Najjar et al. demonstrated that age-related changes of the lens can be predicted more reliably by sHFP with a quadratic model (*r*^2^ = 0.71) than with Scheimpflug imaging (*r*^2^ = 0.46) or with a threshold detection method (*r*^2^ = 0.38) (For technical details of these methods see Najjar et al.^27^). Although the ODI was worse for predicting age-related changes than were measurements by sHFP, lens density at 460 nm measured by the Purkinje image-based technique accurately predicted age-related changes (*r*^2^ = 0.77) with the same accuracy as that of sHFP. Therefore, the Purkinje image-based technique has the potential for rapid measurement of lens density with the same level of accuracy as that of the sHFP method, which can predict age-related changes better than other measurement methods can.

ODI was also highly correlated with lens opacification scale, which is generally used for assessment of lens opacity by ophthalmologists in a clinical setting. This suggests that ODI is an appropriate index for assessment of lens opacity. Moreover, our findings highlight inter-individual differences in ODI, even in subjects with a clinical opacification scale of Grade 0. This suggests that lens opacification can be assessed objectively with a higher resolution than a subjective scale when ODI assessments are used. Although subjective assessment of lens opacity by an ophthalmologist using a slit lamp such as assessment by the Emery-Little classification^43^ or by the lens opacities classification system III (LOCS III)^44^ is widely used in a clinical setting, ophthalmology experience of the observer and accuracy of the slit lamp setting may affect the reliability and reproducibility of the diagnosis^45,46^. ODI measured by our objective method may provide more reliable information on lens opacity than that provided by subjective assessments.

A comparison of lens density spectra measured by the Purkinje image-based technique with and without mydriasis showed that there was no significant main effect of pupil condition. This result suggests that it is not necessary to dilate the pupils for measurements by the Purkinje image-based technique. Clinical subjective assessments using a slit lamp are time-consuming because the pupils need to be dilated for accurate assessments. In contrast, measurements by the Purkinje image-based technique enable evaluation of lens opacity without mydriasis and thus can be easily used for assessments of in a clinical setting.

The estimated lens transmittance was significantly lower in the older group than in the young and middle-aged groups. In contrast, there was no significant difference in lens transmittance between the young group and middle-aged group. These results agree with the results of Najjar’s study^27^ which suggested that lens transmittance decreases gradually with aging and at a higher rate after the age of 60 years (i.e., quadratic changes with aging^26^). Therefore, lens transmittance spectra obtained by our method seem to be reliable for detecting age-related changes of the lens. However, the results for ODI showed that there was a significant difference in lens density between the young group and middle-aged group. Although this discrepancy may be neglected due to the fact that ODI showed quadratic changes with aging as with lens transmittance and the fact that the effect sizes (*η*^2^) of age on lens transmittance and ODI were the same levels (transmittance, *η*^2^ = 0.67; ODI, *η*^2^ = 0.66), we need to determine which is more accurate, raw data of measured lens density or fitted data of estimated lens transmittance.

Measurements by the Purkinje image-based technique and our approach for estimating lens transmittance spectra are expected to be useful in various research fields. It has been reported that the decrease of lens transmittance spectra with aging may affect visual^5,6^ and non-visual functions^7–9^ in various age groups. In those previous studies, however, the detailed relationships between lens transmittance spectra and visual/non-visual functions could not be investigated because there was no method for assessments of transmittance spectra in vivo. By using the present approach, assessments of lens transmittance spectra and estimation of the spectral quality and quantity of light reaching the retina can be performed objectively, easily, rapidly and accurately, and our approach should make some contributions to various research fields which need to information on retinal illuminance such as studies on non-visual lighting effects^47,48^. For non-visual research, for example, relative non-visual photoreception reduction with aging can be estimated from our results for lens transmittance (Fig. 6) and pupil size (Table 1) based on Turner’s calculation^11^. The relative value of non-visual photoreception in our middle-aged group (42.7 ± 4.1 years of age) and older group (58.9 ± 6.7 years of age) with mydriasis, as relative values to young group (27.2 ± 3.7 years of age), were 0.83 and 0.54, respectively. According to Turner’s report, the relative value of non-visual photoreception of 45-year-old and 55-year-old people, as relative values to 25-year-old people, were 0.6 and 0.4, respectively^11^. This discrepancy might have been caused by the large individual variation in lens density^21^, even though there were only slight differences in age and pupil condition. In other words, these results suggest the need to take into account individual differences in lens transmittance in non-visual age-difference studies^7–9,49^.

For assessments of lens opacities in clinical fields, optical coherence tomography (OCT) is becoming increasingly popular for studying the anterior segment (including the cornea, aqueous humor and lens) of the human eye. Some investigators have proposed the use of two-dimensional^50–53^ or three-dimensional^54^ OCT images to grade the scattering produced by the nucleus of the lens and have found a positive correlation with the subjective clinical lens opacification scale using a slit lamp. Recently, Grulkowski et al. demonstrated that a custom-built 3-D OCT scanner enabled volumetric visualization of the detailed structure in the human crystalline lens in vivo and suggested that 3-D OCT is a useful tool for high-resolution evaluation and management of crystalline lens opacities in cataract patients^55^. Chen et al.^52^ and Panthier et al.^56^ demonstrated IOLMaster 700, a widely available 2-D OCT scanner on clinical setting, is a reliable and repeatable method for assessment of cataract compared to LOCS III scale, Scheimpflug imaging or double-pass aberrometry. However, the system for OCT is a relatively complicated optical system with a bulky and costly apparatus, even with the recent low-cost OCT design^57^. In contrast to OCT or conventional techniques (e.g., Scheimpflug imaging^58^ or double-pass aberrometry^59,60^), a compact and low-cost device can be achieved using the Purkinje image-based technique. This is because the optical system in the Purkinje image-based technique consists of only a light source and a camera, and it could be easily miniaturized by various computing platforms such as Raspberry Pi-based system^61,62^. Although the Purkinje image-based technique cannot provide information on the detailed structure in the ocular lens unlike evaluation by OCT, the compact and low-cost device based on our technique allows for the computation of the lens transmittance over the entire visible spectrum and offers a simple investigation tool for visual and non-visual photoreception research.

Our work has some limitations. Firstly, although a compact device can be used for the Purkinje image-based technique as described above, the setup used in the present study was relatively bulky. In addition, the measurement time of our system, approximately 4 s, was slightly slower than that of Johnson’s LAM technique, approximately 2 s^39^. These problems can be solved by using a light source that is compact and can switch the light wavelength at a high speed such as LEDs instead of a xenon arc lamp and/or by using a smaller camera than our CMOS camera. Secondly, the optical density at 430 nm could not be measured in the older group. The lack of information on optical density at 430 nm may provide some inappropriate results for estimation of transmittance spectra. However, the age-related changes of the lens can be sufficiently detected by using the optical densities at 460 nm (*r*^2^ = 0.77) (Fig. 3a). Therefore, it is assumed that transmittance spectra can be estimated even without measurement at 430 nm. Alternatively, a solution for this problem is to increase the intensity of the short wavelength incident light as performed in Johnson’s work^39^, although the risk of blocking the IVth Purkinje image by large pupil contraction is increased. However, if we use a light source in which some single-wavelength lights in the visible region can be switched within approximately 200 ms, that risk may be avoided because the procedure for measurements is completed before the onset of pupil contraction^63^. Finally, the subjects in this study were restricted to subjects without severe cataracts. Although our results showed that the Purkinje image-based technique can be applied to subjects of various ages, it is unclear whether the ocular lens of severe cataract patients can be assessed by our technique. Therefore, we need to investigate whether lens density and transmittance spectra can be assessed in severe cataract patients in order to determine the utility of the Purkinje image-based technique for clinical settings in the future.

In conclusion, our results showed that lens transmittance spectra can be estimated from lens density spectra measured by the Purkinje image-based technique. ODI is an index of lens opacity that enables detection of age-related changes of the lens with high accuracy. Lens densities measured at 460 nm, i.e., at a short wavelength, accurately predicted the increase of lens density with aging. Moreover, ODI was highly correlated with clinical lens opacification scale and is thus a reliable index of lens opacity. In addition, optical density spectra can be measured by the Purkinje image-based technique without pupil dilatation because the results of lens densities did not depend on pupil conditions (non-mydriasis or mydriasis) in any of the age groups. Our findings suggest that our approach can provide objective information on spectral lens density and transmittance rapidly and with a high level of accuracy and it should be useful in both clinical and research settings.

In the future, we expect that our Purkinje image-based system will be improved so as to enable evaluation of not only spectral density and transmittance but also spectral scattering characteristics in the ocular media by applying Santos’s approach^36^. Although the evaluated scattering consists of the combined contributions originating from the cornea, anterior chamber and lens, there is a possibility that the spectral scattering characteristics of the anterior segment can be measured by contrast of the IVth Purkinje image, which is detected easily even in the visible light wavelength region. The improvement of our method would provide more detailed information on optical characteristics (spectral density, transmittance and scattering in the visible region) in the ocular media in vivo.

## Methods

### Participants

Twenty-six adults (ten males and sixteen females) of various ages (age range 22–67 years; mean age ± SD, 40.7 ± 12.8 years) participated in this study. Inclusion criteria were healthy individual, no ocular disease other than cataract, and mild to moderate myopia. Exclusion criteria were any ophthalmological disease with cornea, retina, optic disc, glaucoma, diabetic retinopathy, high myopia, and a history of ocular surgery. Therefore, all of the participants had healthy eyes except cataracts or myopia. All participants were examined by a board-certified ophthalmologist. The participants were divided into three groups by age: a young group including ten participants (20–34 years old; 27.2 ± 3.7 years old), a middle-aged group including nine participants (35–49 years old; 42.7 ± 4.1 years old) and an older group including seven participants (50–70 years old; 58.9 ± 6.7 years old). An oral and paper-based explanation was given before the experiment for all participants. All of the participants gave written informed consent for participation in the study, which was approved by the Ethical Committee of Kyushu University, Japan. The present study was conducted according to the principles of the Declaration of Helsinki.

### Principle of objective measurements of optical density

**Figure 7:**
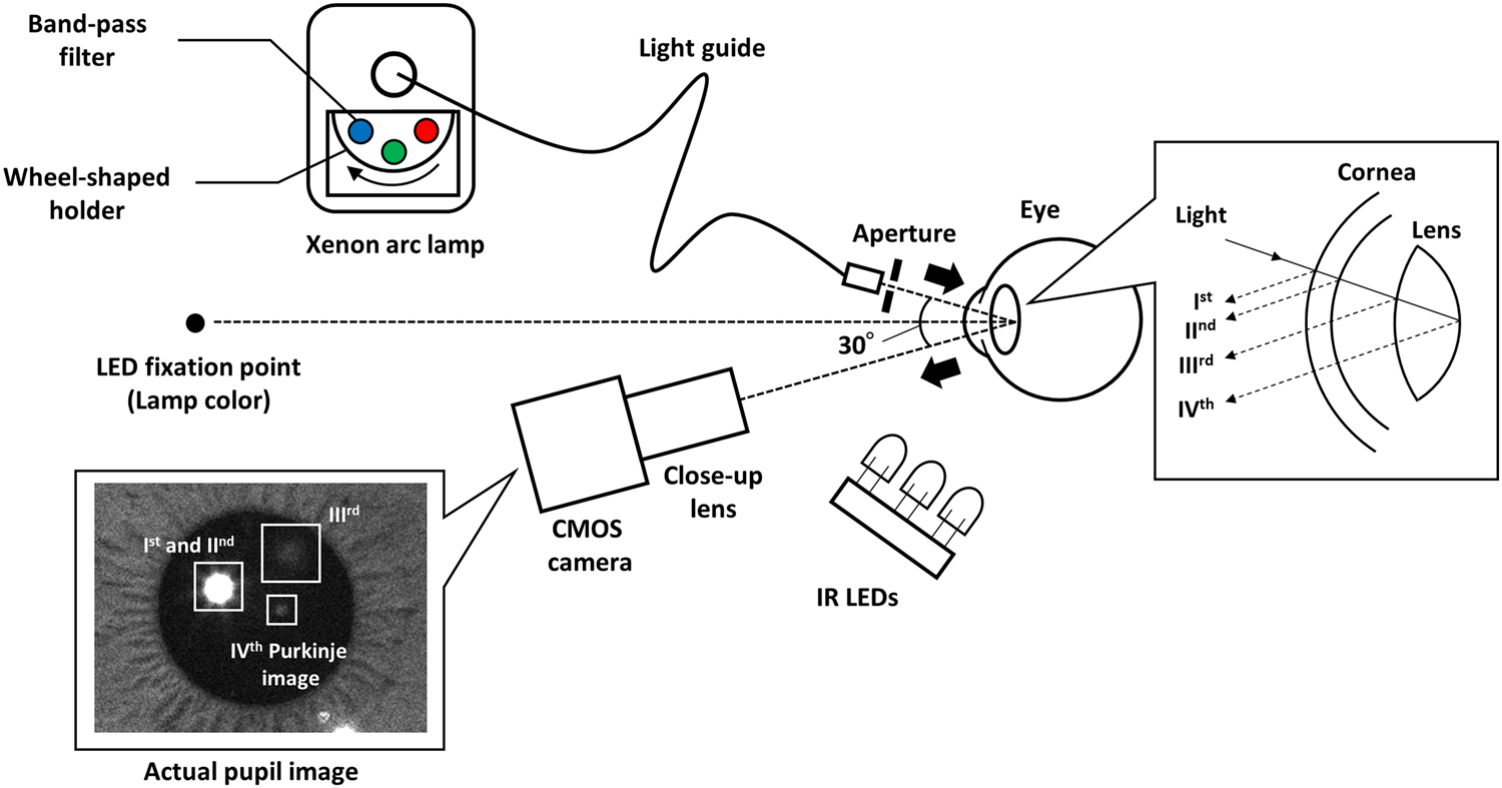
A schematic diagram of the Purkinje image-based experimental setup for objective measurements of human lens density based on the lens absorption monitor (LAM) technique proposed by Johnson et al.^39^.

Figure 7 shows a schematic diagram of the experimental setup. This setup of the Purkinje image-based technique for objective measurements of human lens optical density is based on the lens absorption monitor (LAM) technique proposed by Johnson et al.^39^. Purkinje images are formed by reflections of light sources at different ocular interfaces (air-cornea, cornea-aqueous humor, aqueous humor-lens and lens-vitreous humor interfaces) as shown in Fig. 7. The Ist and IInd Purkinje images are reflection images at the air-cornea and cornea-aqueous humor interfaces, respectively. The Ist and IInd Purkinje images are usually overlapped because the thickness of the cornea is small. The IIIrd Purkinje image, which is a reflection image at the aqueous humor-lens interface, has the largest size, and the IVth Purkinje image, which is a reflection image at the lens-vitreous humor interfaces, is usually slightly smaller in size than the Ist and IInd Purkinje images^64^. Detailed description and actual Purkinje images are beyond the scope of this paper and are provided elsewhere^34,65–67^. Purkinje images have been applied to methods for eye-gaze tracking^37,68,69^, measurements of the alignment of ocular surfaces^64^ and intraocular lenses implanted during cataract surgery^70–72^. Meanwhile, our Purkinje image-based technique enables calculation of the spectral optical density of the anterior ocular media, mainly the crystalline lens, by measurement of the relative intensity of light reflected off the posterior capsule of the crystalline lens (i.e., the IVth Purkinje image) for a range of wavelengths of visible light. The IVth Purkinje image is much easier to locate than the IIIrd Purkinje image, and it can usually be detected without having to dilate a subject’s pupil^39^.

Light from a xenon arc lamp (MAX-301, Asahi Spectra Inc., Japan) was passed through one of eight band-pass filters with peak wavelengths of 430, 460, 470, 480, 500, 520, 540 and 600 nm (MX430–MX600, Asahi Spectra Inc., Japan) and with half-bandwidths of 10 nm, and was incident on the participant’s eye. The corneal irradiances of incident light measured by an illuminance spectrophotometer (CL500A, Konica Minolta Inc., Japan) were 6.0 × 10^–2^ to 7.4 × 10^–2^ W m^−2^ and they were within the safety limitations in ICNIRP guidelines (ISO 15004-2: 2007)^73^. The band-pass filters were fixed to a wheel-shaped holder in the MAX-301, and the band-pass filter through which light passed was switched by rotating the holder. The light that passed through the band-pass filter was led by a light guide to an aperture of 3 mm in diameter. The light emitted from the end of the light guide was diverged, not collimated. The aperture was placed in the tip of light guide to make the light from the light guide a pinpoint source. The distance between the aperture and the participant’s eye was 9 cm. Light exposures were performed in order starting with the short wavelength (430 nm) to the long wavelength (600 nm). Exposure to the eight wavelengths was completed in approximately 4 s. The light reflected from the posterior surface of the crystalline lens was detected by a complementary metal–oxide–semiconductor (CMOS) camera (BFS-U3-32S4M-C, FLIR, United States) with a close-up lens (50 mm C Series VIS–NIR Fixed Focal Length Lens #67-717, Edmund Optics, United States). The sensor size of the CMOS camera was 7.1 × 5.4 mm (Type 1/1.8), and the bit depth of the CMOS camera was 12 bits. The frame rate and the exposure time of the camera were set to 60 fps and 15 ms, respectively. Focal length and numerical aperture of the close-up lens were 50 mm and 0.018, respectively. The horizontal field of view (FOV) and the magnification ratio were 34.9 mm and 0.20, respectively. The angular separation between the illuminant housing and the video camera was fixed at just under 30°. A lamp color LED fixation point was located at a distance of 40 cm from the participant’s eye, and the participants were instructed to stare at the fixation point during measurement. The IVth Purkinje image’s size and intensity (pixel value) and the participant’s pupil diameter were calculated from detected images using the image processing software Image J^74^. To detect clear images of the pupil, the eye was exposed to infrared (IR) LEDs.

Before measurements were performed in each participant, the IVth Purkinje image’s size and intensity at each wavelength were measured in an artificial eye (OEMI-7, Ocular Instruments Inc., United States) in which optical density is constant in the visible light range as a reference. The spectral optical density of the artificial ocular lens was measured by comparing the intensities of the IIIrd and IVth Purkinje images, based on Said and Weale’s procedure, to confirm that it is constant in the visible light range. The spectral optical density $D(\lambda )$ of the participant’s lens was calculated with the following modification of the equation proposed by Said and Weale^33^:

1 $$ \begin{array}{*{20}c} {D\left( \lambda \right) = \frac{1}{2}\left( {\log \frac{{I_{R} \left( \lambda \right)}}{I\left( \lambda \right)} - \log \frac{A\left( \lambda \right)}{{A_{R} \left( \lambda \right)}}} \right),} \\ \end{array} $$

where *λ* means wavelength, $I\left( \lambda \right)$ and $A\left( \lambda \right)$ are the intensity and size of the IVth Purkinje image at each wavelength in the participant’s eye, respectively, and $I_{R} \left( \lambda \right)$ and $A_{R} \left( \lambda \right)$ are the intensity and size of the IVth Purkinje image in the artificial eye, respectively, as a reference. Only the right eye of each participant was evaluated by the Purkinje image-based technique.

The calculated optical densities at each wavelength were transformed into relative values to 600 nm. The area under the curve of relative optical density was defined as an index of lens opacification, optical density index (ODI). The lens transmittance spectrum was derived from $D(\lambda )$ by using the ocular media model proposed by van de Kraats and van Norren^26^.

### Subjective clinical assessment of the lens

Subjective clinical assessment of lens opacification was conducted in a mydriasis condition after measurements by the Purkinje image-based technique. The pupil was dilated with an eye drop containing 0.4% tropicamide. The participant’s anterior eye segment was examined with a slit lamp by an ophthalmologist. Lens opacity was visualized (nuclear and cortical) and subjectively quantified on a six-step validated scale (Grades 0–5) in the Emery-Little classification^43^. Only the right eye of each participant was assessed, as in the case of measurement by the Purkinje image-based technique.

To investigate the effect of pupil size on results of measurement by the Purkinje image-based technique, measurement by the Purkinje image-based technique was performed again in each participant in a mydriasis condition after completion of the clinical assessment.

### Data and statistical analyses

The area under the curve of the optical density spectrum in range from 460 to 600 nm was calculated as the ODI in all participants, although the optical density was actually measured from 430 to 600 nm. This is because there were some older participants for whom the IVth Purkinje image at 430 nm could not be detected due to excessively high optical density.

Statistical analyses were performed with R version 3.4.3 and regression analyses were performed with MATLAB (Math Works). For analysis of the effect of wavelength on the optical density spectrum measured by the Purkinje image-based technique, one-way analysis of variance (ANOVA) was performed independently for each age group. Post hoc analyses were done by using multiple comparisons of two-tailed paired t-tests with modified sequentially rejective Bonferroni (MSRB) correction^75^.

ODIs and areas under the curves of estimated spectral transmittance were compared in the age groups by using one-way ANOVA with multiple comparisons of unpaired t-tests corrected by MSRB. Comparisons of clinical lens opacification scales in the age groups were performed by the Kruskal–Wallis test, and post hoc analyses were done by the Wilcoxon rank sum test with Bonferroni correction.

ODI and clinical lens opacification scale were fitted as a function of age by using a quadratic trend that ignored the linear term ($f = y0 + bx^{2}$) as suggested by van de Kraats and Van Norren^26^. Relationships between opacity indexes of the lens (ODI and clinical lens opacification scale) and age were evaluated by Spearman’s correlation test. The relationship between ODI and clinical lens opacification scale was also evaluated by Spearman’s correlation test.

To investigate the effect of pupil size on optical density measured by the Purkinje image-based technique, mixed repeated measures ANOVA was performed independently for each age group. Pupil condition (non-mydriasis or mydriasis) was used as a between factor, and wavelength was used as a within factor. In the older group, data for five participants excluding two participants for optical density could not be calculated in a mydriasis condition were analyzed. *P* < 0.05 was considered to be statistically significant in all statistical analyses.
